# Is there Evidence for a Continued Benefit for Long‐Term Lecanemab Treatment? A Benefit/Risk Update from Long‐Term Efficacy, Safety and Biomarker Data

**DOI:** 10.1002/alz.092094

**Published:** 2025-01-09

**Authors:** Christopher H. van Dyck, Reisa A Sperling, Shobha Dhadda, David Li, Steven Hersch, Michael C. Irizarry, Lynn D Kramer

**Affiliations:** ^1^ Alzheimer’s Disease Research Unit, Yale School of Medicine, New Haven, CT USA; ^2^ Brigham and Women’s Hospital and Department of Neurology, Massachusetts General Hospital, Harvard Medical School, Boston, MA USA; ^3^ Eisai Inc., Nutley, NJ USA; ^4^ Deep Human Biology Learning (DHBL), Eisai Inc., Nutley, NJ USA

## Abstract

Lecanemab, a humanized IgG1 monoclonal antibody that binds with high affinity to amyloid‐beta (Aβ) protofibrils, was formally evaluated as a treatment for early Alzheimer’s disease in a phase 2 study (Study 201) and the phase 3 Clarity AD study. These trials both included an 18‐month, randomized study (core) and an open‐label extension (OLE) phase where eligible participants received open‐label lecanemab for up to 30 months to date. Clinical (CDR‐SB, ADAS‐Cog14, and ADCS‐MCI‐ADL), biomarker (PET, Aβ42/40 ratio, and ptau181) and safety outcomes were evaluated. Results demonstrated that lecanemab substantially reduced markers of amyloid and significantly slowed clinical decline on multiple measures of cognition, function, and quality of life in early AD at 18 months and continued for 30 months to date. Lecanemab was associated with amyloid‐related imaging abnormalities (ARIA) and infusion reactions, tending to occur early in treatment. Although lecanemab has established a clear benefit/risk benefit for 18 months of treatment, the evidence for continued treatment beyond 18 months has yet to be firmly established. In an effort to evaluate the current evidence base for longer‐term dosing, this presentation will summarize the latest clinical and biomarker results from the lecanemab Clarity AD study, including the latest efficacy and safety data out to at least 30 months. We will share details on how lecanemab has demonstrated the ability to slow tau spread in different brain regions of individuals with early Alzheimer’s disease and discuss the relevance of these data on continued treatment. Evaluations of clinical and biomarker outcomes by participants considered as having a ‘delayed start’ (core:placebo followed by OLE:lecanemab) versus those with an ‘early start’ (core:lecanemab followed by OLE:lecanemab) cohorts will be presented and discussed. Finally, the overall case for the need and benefit of early initiation and continued dosing, considering mechanistic, pharmacology, biomarker and clinical results, will be discussed.

